# In silico comparison of Iranian HIV -1 envelop glycoprotein with five nearby countries

**Published:** 2016-06

**Authors:** Maryam Ghafari, Mandana Behbahani, Hassan Mohabatkar

**Affiliations:** Department of Biotechnology, Faculty of Advanced Sciences and Technologies, University of Isfahan, Isfahan, Iran

**Keywords:** Amino acid composition, Envelope glycoprotein, HIV-1, Physical and chemical properties

## Abstract

HIV-1 envelope (env) glycoprotein mediates an important role in entry of the virus into the susceptible target cells. As env glycoprotein of HIV-1 is highly variable in the different geographical regions, in the present study, different properties of this protein in Iran are compared with five nearby countries. The sequences of HIV-1 env glycoproteins of Iran, Afghanistan, Russia, Turkey, Pakistan and Saudi Arabia databases were collected from databases. Amino acid composition and physical and chemical properties of the proteins from these countries were studied using Protparam and COPid tools. Receiver-operating characteristic (ROC) curve analysis and Support Vector Machine (SVM) were used to evaluate association between the properties of HIV-1 env glycoprotein of Iran with five nearby countries. The results verify that amino acid composition and four physical and chemical properties (molecular weight, isoelectric point, Aliphatic Index, and grand average of hydropathicity) of HIV-1 env protein in Iran and Russia were not significantly different. In conclusion, the results indicate that in silico techniques provide valuable information for comparing HIV-1 envelop glycoprotein in different geographical locations.

## INTRODUCTION

HIV-1 is one the most important pathogens and causes the majority of HIV infections globally [1, 2]. HIV-1 is a member of Retroviridae and is sorted into three typical groups; group M (main), group O (outliner), and group N (non-M/non-O) [3, 4]. The majority of the infection is caused by group M which is divided into 9 different subtypes symbolized A, B, C, D, F, G, H, J and K [5, 6]. Genome of HIV is composed of two positive strands RNA which are packaged in a protein capsid and surrounded by a lipid env [7]. HIV-1 env glycoprotein is essential for entry of the virus into the cell by binding to the specific receptors on the surface of the target cells [8]. HIV-1 env glycoprotein has proved to be useful to study of variation in HIV strains by a number of approaches [4]. Some of these approaches are based on amino acid sequences, amino acid composition and physical and chemical properties of proteins. Physical and chemical properties of viral proteins are widely used to predict various aspects of proteins such as molecular weight, isoelectric point, instability index, aliphatic index and grand average of hydropathicity (GRAVY) [9]. Several researchers have worked on evolutionary patterns of HIV-1 envelop group M in African, Asia, Europe and America [10]. In addition, phylogenetic analysis of HIV-1 env glycoprotein of different Asian countries such as India, Bangladesh, Cambodia, China and Japan were studied [11]. But the diversity of HIV-1 envelops in Iran and some nearby courtiers have not been investigated yet. In the present study, the Amino acid composition and some physical and chemical properties of HIV-1 env proteins of different subtypes in Iran and five nearby countries are studied.

## MATERIALS AND METHODS

**Data collection: **Amino acid sequences of HIV-1 env protein from six different countries (Iran, Pakistan, Russia, Saudi Arabia, Afghanistan and Turkey) were collected from NCBI (http://www.ncbi.nlm.gov/protein). Two hundred sixty two amino acid sequences from Iran and 752 sequences from five nearby countries (349 Pakistan, 280 Russia, 62 Saudi Arabia, 34 Afghanistan and 27 Turkey) were studied. Redundant sequences (more than 95% similarity) were removed from our dataset by CD-HIT. After running CD-HIT, the number of sequences existing in the dataset in Iran, Pakistan, Russia, Saudi Arabia, Afghanistan and Turkey were 123, 123, 50, 26, 12 and 30 respectively. In the next step, all nucleotide sequences of these 6 groups were also collected from NCBI.

**Server and tools:**

*Context-based Modeling for Expeditious Typing (COMET)*: All nucleotide sequences of these HIV-1 env genes of the 6 countries were analyzed using the Calibrated Population Resistance (CRP) subtyping tool COMET to predict different subtypes. COMET (v. 0.2) is a reliable tool to predict HIV-1/2 subtypes [12]. This tool is available at (http://comet.retrovirology.lu) and uses Prediction Partial Matching (PPM) compression algorithm.

ProtParam tool: ProtParam is a server which computes different physical and chemical parameters of a protein [13]. The web-server is available at http://web.expasy.org/protparam. Four characteristics (molecular weight, isoelectric point, GRAVY and aliphatic index) of HIV-1 env glycoprotein from Iran and five nearby countries were evaluated by ProtParam.

Composition protein identification: The amino acid sequences of env glycoprotein from these six countries were analyzed using Composition Based Protein Identification (COPid). COPid is a server which analyzes composition of various types of amino acids [14]. The COPid web-server is available at http://www.imtech.res .in/raghava/ COPid. This server helps the researchers to elucidate the function of a protein and generate a phylogenetic tree from its composition.

**Statistical Analysis: **The data were evaluated by Receiver Operating Characteristic (ROC) curve and Molegro Data Modeller (MDM). ROC curve is a tool for organizing classifiers and visualizing their performance. ROC curves are usually used in machine learning and data mining research [15]. ROC server can calculate accuracy (ACC) and compute association between the properties of HIV-1 env glycoprotein in Iran and five nearby countries near Iran. ACC is a factor to differentiate positive and negative classes of data. When ACC value is more than 0.8, it means that the difference between two classes is significant. ACC is calculated using the following formula:

ACC = Σ True positive + Σ True negative/ Σ Total population

MDM is a cross-platform application for data mining and data visualization. MDM generates regression and classification models by partial least squares, neural networks and support vector machines [16]. SVMs are supervised learning algorithms which are broadly used in classification or regression problems [17].

## RESULTS

The result of HIV-1 subtyping in Iran, Russia, Turkey, Saudi Arabia, Pakistan and Afghanistan are summarized in Table 1. The results showed that subtype A was the dominant subtype in Iran, Russia and Pakistan. But dominant subtypes in Saudi Arabia, Turkey and Afghanistan were C, B and AD, respectively.

**Table 1:** COMET subtyping tool Results I: Iran A: Afghanistan P: Pakistan T: Turkey R: Russia SA: Saudi Arabia

Countries	A	AD	AE	AG	B	C	D
I	46%	20%	-	-	26%	8%	-
A	-	93%	7%	-	-	-	-
P	95%	3%	2%	-	-	-	-
T	12%	-	-	-	72%	-	3%
R	70%	-	-	15%	15%	-	-
SA	3.1%	-	-	4%	20%	37%	5%

Analysis of ProtParam results using ROC curve and SVM are presented in Tables 2 and 3. Table 2 shows that ACC values of GRAVY, aliphatic index, isoelectric point and molecular weight between Iran and Russia were 0.56, 0.65, 0.62 and 0.65 respectively. But ACC values between Iran and four other countries were more (Table 2). As the results of ROC analysis between Iran and Russia were less than 0.8, physicochemical properties of viral env protein between these two countries were not significantly different. The results of MDM analysis approved our previous results (Table 3), because values of four parameters such as molecular weight, isoelectric point, aliphatic Index and GRAVY between Iran and Russia were less than 6.4 and the values between Iran and four other countries were more than 0.7. The results of ROC curve and MDM analyses showed that physicochemical properties of HIV-1 env protein in Iran and Russia were not significantly different.

**Table 2:** ROC Analysis of ProtParam Results I: Iran A: Afghanistan P: Pakistan T: Turkey R: Russia SA: Saudi Arabia

Classifier	I-T	I-R	I-A	I-SA	I-P
GRAVY	0.68	0.62	0.91	0.89	0.83
Aliphatic Index	0.98	0.65	0.94	0.97	0.92
PI	1.00	0.56	0.89	0.87	0.97
MW	0.73	0.65	0.93	0.80	0.84

**Table 3:** Performance of MDM I: Iran A: Afghanistan P: Pakistan T: Turkey R: Russia SA: Saudi Arabia

Classifier	I-T	I-R	I-A	I-SA	I-P
GRAVY	0.77	0.50	0.91	0.89	0.81
Aliphatic Index	0.97	0.64	0.93	0.98	0.89
PI	1.00	0.50	0.91	0.87	0.92
MW	0.80	0.61	0.90	0.80	0.84

The results of ROC curve analysis for amino acid composition of six databases are been shown in Table 4. The ACC values of 17 amino acids out of 20 amino acids between Iran and Russia were less than 70%. These amino acids were Asp, Ala, Glu, Phe, Ile, His, Gly, Leu, Asn, Lys, Arg, Pro, Gln, Thr, Val, Trp and Tyr. However ACC values between Iran and four other countries were significantly different.

The results of Molegro analysis for amino acid composition of six databases are shown in Table 5. The ACC values of 17 amino acids out of 20 amino acids between Iran and Russia databases were less than 0.7. The ACC of Cys, Met, and Ser between Iran and Russia were more than 0.7.

**Table 4:** ROC analysis results of 20 amino acids of six databases I: Iran A: Afghanistan P: Pakistan T: Turkey R: Russia SA: Saudi Arabia

Classifier	I-T	I-R	I-A	I-SA	I-P
Asp	0.85	0.62	0.90	0.71	0.92
Cys	0.98	0.74	0.90	0.95	0.96
Ala	0.81	0.66	0.90	0.84	0.83
Glu	0.91	0.56	0.82	0.71	0.83
Phe	0.99	0.67	0.90	0.91	0.99
Ile	0.84	0.56	0.86	0.70	0.84
His	0.93	0.64	0.82	0.87	0.90
Gly	1.00	0.65	0.85	0.77	1.00
Leu	1.00	0.64	0.96	0.96	1.00
Met	0.85	0.71	0.90	0.86	0.81
Asn	0.89	0.60	0.96	0.89	0.84
Lys	0.81	0.56	0.75	0.76	0.80
Arg	0.97	0.59	0.82	0.86	0.97
Pro	0.99	0.63	0.83	0.96	1.00
Ser	0.90	0.75	0.78	0.76	0.83
Gln	1.00	0.60	0.82	0.95	0.98
Thr	0.96	0.62	0.91	0.98	0.99
Val	0.95	0.56	0.80	0.69	0.86
Trp	1.00	0.66	0.88	0.96	1.00
Tyr	0.93	0.60	0.88	0.80	0.91

**Table 5:** Molegro analysis results of 20 amino acids of six databases I: Iran A: Afghanistan P: Pakistan T: Turkey R: Russia SA: Saudi Arabia

Classifier	I-T	I-R	I-A	I-SA	I-P
Asp	0.88	0.54	0.91	0.71	0.88
Cys	0.98	0.74	0.91	0.94	0.94
Ala	0.82	0.61	0.91	0.82	0.87
Glu	0.89	0.53	0.91	0.71	0.85
Phe	0.97	0.60	0.91	0.89	0.98
Ile	0.82	0.56	0.91	0.71	0.81
His	0.85	0.50	0.91	0.83	0.89
Gly	1.00	0.62	0.91	0.76	1.00
Leu	0.99	0.65	0.90	0.95	1.00
Met	0.81	0.66	0.91	0.83	0.85
Asn	0.87	0.69	0.90	0.87	0.83
Lys	0.82	0.52	0.91	0.70	0.80
Arg	0.95	0.54	0.91	0.88	0.95
Pro	0.97	0.50	0.91	0.96	1.00
Ser	0.86	0.72	0.91	0.72	0.82
Gln	0.99	0.50	0.91	0.95	0.97
Thr	0.93	0.65	0.90	0.97	0.98
Val	0.93	0.50	0.91	0.75	0.85
Trp	1.00	0.65	0.89	0.96	1.00
Tyr	0.93	0.58	0.91	0.73	0.89

## DISCUSSION

In the present study, association between the properties of HIV-1 env glycoprotein in Iran and five nearby countries are studied. According to literature, the most variations in HIV-1 are related to the env glycoproteins gp41 and gp120 sequences [18, 19]. In the large number of cases, phylogenetic clustering of HIV-1 isolates is based on the differences in env genes nucleotide sequences [20, 21]. HIV-1 env proteins of different subtypes and sub-subtypes can vary in more than 30% of their amino acids [22, 23]. In recent decades, several phylogenetic classifications are proposed on the HIV-1 env glycoprotein in Asian, African, European and American countries. In 2006, Ahn and Son reported that codon usage patterns among the HIV-1 env proteins of different subtypes may be a useful method to predict the evolutionary patterns of pandemic viruses [10]. In 2007, Singh and Seth, analyzed amino acid sequences of HIV env Protein by means of Clustal X software and found the association between the sequences from different Asian countries [11]. Our results also demonstrate that amino acid composition and four physical and chemical properties of HIV-1 env protein in Iran and Russia were less than 0.6. According to literature when the ACC values are less than 0.6, it means that differences between positive and negative classes of data are not significant [24]. Therefore, viral env proteins in Iran and Russia were not significantly different. Physicochemical properties between Iran and four different countries Turkey, Afghanistan, Pakistan and were significantly different. The result also showed that subtype A is dominant in Iran and Russia. Some researchers have reported that subtype A can circulate at high rate among intravenous drug users and the most probable way for HIV-1 subtype A introduction into Iran is through Former Soviet Union countries[25,26,27]. These observations indicated that in silico properties of HIV-1 env protein in Iran and Russia are similar.
